# Robotic Surgery Is a Safe Treatment in Very Elderly Patients with Resectable Lung Cancer

**DOI:** 10.3390/jcm14124314

**Published:** 2025-06-17

**Authors:** Pierluigi Novellis, Riccardo Di Fonzo, Edoardo Bottoni, Veronica Maria Giudici, Domenico Pontillo, Piergiorgio Muriana, Elisa Dieci, Roberto Ferrara, Alessandra Bulotta, Giuseppe Marulli, Gianluca Perroni, Giulia Veronesi

**Affiliations:** 1 Department of Thoracic Surgery, IRCCS San Raffaele Scientific Institute, 20132 Milan, Italy; muriana.piergiorgio@hsr.it (P.M.); dieci.elisa@hsr.it (E.D.); veronesi.giulia@hsr.it (G.V.); 2 Vita—Salute San Raffaele University, 20132 Milan, Italy; ferrara.roberto@hsr.it; 3 IRCCS Humanitas Research Hospital, Via Manzoni 56, Rozzano, 20089 Milan, Italy; edoardo.bottoni@cancercenter.humanitas.it (E.B.); veronica.giudici@cancercenter.humanitas.it (V.M.G.); marulli.giuseppe@hsr.it (G.M.); 4 Department of Anaesthesia and Intensive Care, IRCCS San Raffaele Scientific Institute, 20132 Milan, Italy; pontillo.domenico@hsr.it; 5 Department of Oncology, IRCCS San Raffaele Scientific Institute, 20132 Milan, Italy; bulotta.alessandra@hsr.it; 6 Department of Biomedical Sciences, Humanitas University, Via Rita Levi Montalcini 4, Pieve Emanuele, 20072 Milan, Italy; 7 Thoracic Surgery Unit, IRCCS Ospedale Sacro Cuore Don Calabria, Negrar, 37024 Verona, Italy; drgianlucaperroni@gmail.com

**Keywords:** lung cancer, robotic-assisted thoracic surgery, elderly patients

## Abstract

**Background:** Lung cancer represents a significant health concern, particularly among the elderly population. With global life expectancy increasing, the number of very elderly patients is rising. Robotic-assisted thoracic surgery (RATS) offers potential advantages over both traditional and video-assisted thoracoscopic surgery (VATS). This study aims to evaluate the feasibility and safety of RATS in very elderly patients (VEP) diagnosed with lung cancer. **Methods**: This retrospective study included patients who underwent major lung resections using RATS between 2015 and 2022 at two specialized centers. Patients were divided into very elderly patients (VEP, ≥80 years) and non-elderly patients (NEP, <80 years). Demographic, clinical, and surgical data were analyzed. Propensity score matching (PSM) at a 1:3 ratio was performed using clinically relevant variables that were significantly different at baseline to balance the two groups. **Results**: This study included 340 patients: 28 VEP and 312 NEP. Before PSM, VEP had higher ASA scores, more advanced disease stages, and increased comorbidities. Despite these differences, postoperative outcomes were comparable. Complications occurred in 42.9% of VEP and 29.8% of NEP (*p* = 0.16), but grade III complications were observed in 14.3% of VEP and 6.4% of NEP (*p* = 0.12), and grade IV complications were observed in 0% of VEP and 0.9% of NEP (*p* = not estimable). The mean hospital stay was 4 days in both groups (*p* = 0.99). Even after PSM (26 VEP vs. 71 NEP), complications, hospital stay, and 90-day mortality (3.9% in VEP, 0% in NEP) were similar. Multivariable analysis identified reduced FEV1 as a predictor of complications, while pathological stage I and lobectomy were associated with a decreased risk of complications, both before and after PSM. **Conclusions**: RATS is a safe and feasible option for selected very elderly patients with lung cancer, yielding outcomes comparable to younger patients.

## 1. Introduction

With almost 2.5 million new cases and over 1.8 million deaths worldwide, lung cancer is the leading cause of cancer morbidity and mortality in 2022, responsible for close to one in eight (12.4%) cancers diagnosed globally and one in five (18.7%) cancer deaths [1]. The incidence of cancer escalates with age, with over half of all cases occurring in patients aged 65 and older [2]. Specifically, 14% of those diagnosed with lung cancer are aged 80 or above, referred to as the “very elderly” population [3]. With global increases in life expectancy, the demographic of very elderly individuals expanded from 53 million to 143 million worldwide between 1990 and 2019. Projections by the United Nations World Population Prospects provides that there will be 265 million persons aged 80 years or older in the mid-2030s [4].

The management of very elderly patients in thoracic surgery presents unique challenges, including a heightened risk of postoperative complications such as supraventricular arrhythmia, pneumonia, respiratory failure, and increased mortality rates compared to younger cohorts [5]. These challenges are attributable to both physiological and pathological alterations associated with aging, such as declines in FEV1 and DLCO values—particularly notable after the age of seventy [6,7]—and increased prevalence of Chronic Obstructive Pulmonary Disease (COPD) and Heart Failure (HF) [8,9].

Robotic-assisted thoracic surgery (RATS) has been shown to reduce morbidity, mortality, and length of stay (LOS) compared to open surgery [10]. Comparatively, when RATS is evaluated against video-assisted thoracoscopic surgery (VATS), long-term stage-specific survival rates following RATS lobectomy are similar to those observed with VATS [11]. Additionally, RATS demonstrates reduced 30-day mortality, albeit with longer operative durations [12]. Furthermore, RATS is associated with lower conversion-to-open rates and 30-day complication rates compared to VATS [13]. While RATS generally entails longer mean operative times and higher costs—primarily due to the expense of robotic disposable instruments—a reduction in LOS often offsets these additional costs [14,15,16]. As we observed in a previous study, the cost of robotic surgery is 13.5% higher than VATS, the total expenses are 18% lower than the reimbursement rates of health services in Italy; thus it is sustainable [17].

With the current evidence regarding the application of RATS in the treatment of lung cancer in very elderly patients, there are some gaps in knowledge. When comparing postoperative outcomes of RATS and thoracoscopic (VATS) lobectomy with open surgery performed in octogenarian patients, it was found that VATS and RATS were associated with shorter length of stay (1 and 2 days, respectively). Moreover, RATS also had fewer postoperative complications when compared to open surgery in octogenarians [18]. Another recent study has supported the safety and feasibility of robotic lobectomy in elderly patients, highlighting the predictive role of FEV1 and nodal disease in determining postoperative outcomes [19]. In this study, however, the age limit was defined as 75 years. The objective of our study was to confirm the feasibility and safety of RATS in patients aged 80 years or more defined as very elderly patients (VEP) with suspected or confirmed lung cancer by comparing postoperative and oncological outcomes with those from patients younger than 80 years, herein referred to as non-elderly patients (NEP).

## 2. Materials and Methods

### 2.1. Patients Selection

All patients who underwent major lung resection (segmentectomy, lobectomy, bilobectomy, or pneumonectomy) performed in two referred centers (IRCCS San Raffaele Scientific Institute and IRCCS Humanitas Research Hospital, Milan, Italy) using robotic system from 2015 to 2022 were included in this study. This study was conducted in accordance with the Declaration of Helsinki and approved by the Ethics Committee (Lombardy Territorial 1 Ethics Committee) of IRCCS Ospedale San Raffaele (protocol code CET 234-2024 and date of approval 19 June 2024).

Eligibility criteria included patients aged 18 years or older at the time of surgery, with suspected or confirmed diagnosis of lung cancer, eligible for surgery based on their ASA score and assessments of cardiac, kidney, liver, and respiratory function and without the presence of extrapulmonary metastases. Patients who could not undergo surgery as they did not meet the conditions outlined above were excluded.

Patients were characterized according to demographic variables, including age, sex, smoking history (never, former, and current smokers), clinical variables, comorbidities, previous neoadjuvant therapy, forced expiratory volume in 1 s (FEV1), diffusing capacity of the lungs for carbon monoxide (DLCO), and clinical and pathological stage (I, II, III, and IV), according to the TNM staging system (8th ed) [20]. Patients were divided into two groups: 80 years and older (case) and younger than 80 years (control).

### 2.2. Outcomes

The primary endpoint of this study was to assess the complications. The secondary endpoints were to compare the complications according to the Clavien–Dindo classification [21] to analyze postoperative stay and the mortality rate of robotic resection in VEP with suspected or confirmed lung cancer in comparison with NEP.

### 2.3. Surgical Technique and Follow-Up Protocols

All the surgical procedures were performed using a Da Vinci Xi Surgical Robot (Intuitive Surgical, Inc., Santa Clara, CA, USA). In robotic anatomical lung resection (segmentectomy, lobectomy, bilobectomy, or pneumonectomy), the patient was positioned in lateral decubitus and single-lung anesthesia is induced via a double lumen endotracheal tube. The four-arm Da Vinci technique includes three ports and a utility incision, without routine CO_2_ insufflation [11]. The first 3 cm incision is performed at the fourth or fifth intercostal space anteriorly (utility incision), the second 1 cm incision is performed at the level of the seventh–eighth intercostal space in the midaxillary line and is used to insert a 30-degree stereoscopic camera. The third 8 mm incision is performed at the eighth intercostal space in the posterior axillary line for a robotic arm. The last trocar is inserted posteriorly in the auscultatory triangle in the seventh intercostal space. This incision is used to retract the lung and better expose the operating field. Postoperatively, all patients underwent daily chest radiographs and routine blood tests during hospitalization. A further chest X-ray was performed after chest drain removal to confirm adequate lung re-expansion. Approximately 30 days after surgery, all patients were re-evaluated in an outpatient setting, where they presented with a recent chest radiograph and updated blood tests to assess their clinical recovery.

### 2.4. Statistical Analysis

Statistical analysis was performed using SAS (version 9.4, SAS Institute Inc., Cary, NC, USA), and R (version 4.4.2, R Foundation for Statistical Computing, Vienna, Austria). Dichotomous and categorical variables were presented as absolute frequencies and percentages. Differences between the two groups were tested using the Chi square test or Fisher’s exact test, as appropriate. The normality of continuous variables was assessed using the Shapiro–Wilk test: if normally distributed, data were reported as mean ± standard deviation and differences between groups were tested with the *t*-test; if not normally distributed, data were presented as median and interquartile range, and differences between groups were tested with the Wilcoxon Mann–Whitney test.

Between-group differences for the primary and secondary outcomes were reported as relative risks (RR) or mean differences with their 95% confidence intervals (CI), for dichotomous and continuous variables, respectively. We performed a sensitivity analysis to account for potential confounding by surgical procedure type. A log-binomial regression model was used to estimate the adjusted relative risk (RR) and 95% confidence interval (CI) for the association between the study group (VEP vs. NEP) and the primary outcome, including resection type as categorical covariates (segmentectomy, lobectomy, pneumonectomy, and other resections, including bilobectomy and sleeve bronchoplasty).

For the primary outcome, univariate logistic regression models were used to estimate associations and identify potential predictors with a *p*-value threshold of 0.2. Multivariable stepwise regression models were then applied to adjust for confounding variables, with a significance level set at 0.05.

### 2.5. Propensity Score Matching

To minimize confounding and improve comparability between groups, a propensity score matching (PSM) approach was employed. The propensity score was estimated using a logistic regression model including the following variables selected a priori based on clinical relevance and potential for confounding: sex, tumor size, and number of comorbidities.

Given the small number of exposed patients (*n* = 28) and the availability of a large control pool, a 1:3 nearest-neighbor matching without replacement was performed. An exact match was enforced for gender. This matching ratio was selected to enhance statistical power while preserving covariate balance. Balance between groups was assessed using standardized mean differences (SMD), with values below 0.1 considered indicative of acceptable balance. The final matched dataset was used for all outcome analyses.

## 3. Results

A total of 340 patients were included in this study: 312 patients were NEP and 28 patients were VEP. After PSM, 97 patients, 26 patients in the VEP group, and 71 patients in the NEP group were well matched by a 1:3 PSM algorithm. The demographic, clinical, and surgical data are presented in Table 1.

The median age of the entire population at the time of surgery was 69 years (IQR 63–75). In the VEP group the median age was 81 years (IQR 80–83) while in the NEP group was 68.8 (62–73.9). In the VEP group we observed a higher ASA score, higher clinical stage of the disease, larger pathological mean size of the tumor, higher rates of comorbidities, and higher percentages of smokers and former smokers. All patients enrolled in this study and subsequently followed up underwent radical surgical resection. After PSM there were no statistically significant differences in demographic, clinical, and surgical characteristics between the two groups.

Complications occurred in 12 (42.9%) patients of the VEP and in 93 (29.8%) of the NEP group (RR: 1.43, 95% CI: 0.91–2.7; *p* = 0.16). For the secondary outcomes, no statistically significant differences were observed concerning the onset of different grades of complications according to the Clavien–Dindo classification, before and after PSM. The mean hospital stay was 4 (3–5) days for both groups (RR 0.001, 95% CI: −1.11 to 1.12; *p* = 0.99). The 30-day mortality was zero in either group (*p* = not estimable). The 90-day mortality was 1 (3.6%) in the VEP group and 0 (0%) in the NEP group (*p* = not estimable). After PSM there were no statistically significant differences in the outcomes between the two groups (Table 2a). We performed a sensitivity analysis for primary outcomes adjusted for surgical procedure types. The results are shown in Table 2b and reinforce the robustness and internal validity of our findings.

Before PSM, a univariate logistic regression analysis was performed to identify potential predictors of postoperative complications. History of smoking emerged as a strong predictor, with current smokers having a significantly higher risk of complications (OR: 4.06, 95% CI: 1.83–9.01; *p* = 0.0006), and former smokers also showing increased risk (OR: 3.34, 95% CI: 1.53–7.3; *p* = 0.0025). Additionally, both FEV1 (OR: 0.97, 95% CI: 0.96–0.99; *p* = 0.0001) and DLCO (OR: 0.98, 95% CI: 0.96–0.99; *p* = 0.0071) were identified as significant predictors, with lower values correlating with increased complication rates. The number of comorbidities trended toward significance (OR: 1.27, 95% CI: 0.99–1.63; *p* = 0.059), suggesting a potential impact on outcomes. After PSM, smoking history lost its predictive value, with both current (OR: 1.38, 95% CI: 0.57–3.38; *p* = 0.48) and former smokers (OR: 1.33, 95% CI: 0.58–3.07, *p* = 0.50) showing no significant association. Among functional parameters, FEV1 remained a significant predictor (OR: 0.97, 95% CI: 0.95–0.99; *p* = 0.024), while DLCO did not retain statistical significance (*p* = 0.24). Notably, ASA score emerged as a significant variable post-matching: patients with ASA score I had a lower risk (OR: 0.36, 95% CI: 0.14–0.92; *p* = 0.032) of developing complications, while patients with ASA score III had a higher risk (OR:2.65, 95% CI: 1.12–6.28; *p* = 0.026). Patients treated with lobectomy had a lower risk of developing complications with respect to patients treated with segmentectomy, pneumonectomy, or other anatomical surgical resections (OR: 0.33, 95% CI: 0.12–0.85, *p* = 0.022). Results of the univariate analysis before and after PSM are presented in Table 3.

In the multivariate logistic regression model, after adjusting for confounding variables, FEV1 remained an independent predictor of complications (OR: 0.98, 95% CI: 0.97–0.99; *p* = 0.002), reinforcing the importance of preoperative respiratory function assessment. Smoking history showed a significant association, with never smokers exhibiting a significantly lower risk of complications (OR: 0.36, 95% CI: 0.17–0.78; *p* = 0.01). Results of the multivariate logistic regression are shown in Table 4.

In the multivariate analysis after PSM, initial pathological stage (Stage I) was associated with lower risk of complications (OR: 0.23, 95% CI: 0.08–0.66, *p* = 0.006). Moreover, lobectomy was associated with a decreased risk of complication (OR: 0.29, 95% CI: 0.09–0.91; *p* = 0.033), while FEV1 continued to demonstrate a protective effect in the multivariate analysis after PSM (OR: 0.97, 95% CI:0.95–1.00; *p* = 0.025). Results of the multivariate logistic regression are shown in Table 5.

## 4. Discussion

As the global population ages, the incidence of age-related diseases, including cancer, is increasing at an unprecedented rate [2]. Lung cancer remains one of the most prevalent and lethal malignancies worldwide, with a significant proportion of cases occurring in the elderly. According to recent statistics, over 50% of lung cancer diagnoses are made in patients aged 65 and older, with a notable 14% in those aged 80 and above [3]. This demographic shift towards an aging population necessitates a re-evaluation of surgical approaches to ensure effective and safe treatment for elderly patients.

The management of lung cancer in elderly patients poses unique challenges due to the interplay of age-related physiological changes, comorbidities, and decreased functional reserves. Traditional open thoracotomy, while effective, is associated with significant morbidity and prolonged recovery [10]. Consequently, there has been growing interest in minimally invasive surgical techniques that can reduce perioperative risks and improve outcomes in elderly patients [19].

Robotic-assisted thoracic surgery (RATS) has emerged as a valid alternative to conventional open surgery and video-assisted thoracoscopic surgery (VATS). The precision and control afforded by robotic systems can potentially mitigate the complications associated with traditional surgical methods, offering benefits such as reduced pain, shorter hospital stays, and quicker return to baseline activities. Recent data support the growing feasibility and safety of minimally invasive lobectomies in elderly patients. In a large retrospective study using the Premier Healthcare Database, Sarkaria et al. compared perioperative outcomes of open, video-assisted thoracoscopic (VATS), and robotic-assisted lobectomy (RL) in 1849 octogenarians (≥80 years) who underwent elective lung resection between 2011 and 2015. After propensity score matching, both VATS and RL were associated with significantly shorter hospital stays and higher rates of discharge to home compared to open surgery. Notably, robotic-assisted lobectomy showed a significantly lower overall complication rate, particularly regarding postoperative bleeding and transfusion requirements (*p* = 0.0249), suggesting a potential advantage over traditional thoracotomy in this high-risk population [18].

In addition, a monocentric retrospective study by Gallina et al., evaluated perioperative outcomes in 103 patients aged over 75 undergoing robotic lobectomy. The analysis revealed that reduced predicted postoperative FEV1 and FVC were significantly associated with increased risk of complications (*p* = 0.04), and pathological upstaging emerged as the only independent predictor in logistic regression. Interestingly, the kind of lymphadenectomy was not associated with worse outcomes [19].

Considering the progressive aging of the global population and the growing incidence of lung cancer among older individuals, we chose to stratify our cohort using 80 years as the age threshold. This cut-off reflects a well-recognized definition of the “very elderly” and is supported by recent demographic data projecting a substantial rise in the number of individuals aged ≥ 80 years over the coming decades [4]. As lung cancer is increasingly diagnosed in this age group, understanding the safety and efficacy of surgical interventions in octogenarians has become a matter of pressing clinical relevance. Our decision to compare postoperative outcomes between patients aged ≥ 80 and those < 80 years was therefore motivated by the need to generate evidence applicable to contemporary thoracic surgical practice. This stratification allowed us to specifically assess whether age-related physiological decline and comorbidity burden significantly impact the risk profile of robotic-assisted thoracic surgery (RATS), or whether, in appropriately selected patients, chronological age alone should not preclude surgical treatment. To strengthen the validity of this comparison and reduce the impact of confounding variables, we applied propensity score matching (PSM), balancing the two groups for relevant clinical and functional parameters that may influence postoperative risk.

Our study provides evidence supporting the feasibility and safety of RATS in selected very elderly patients (VEPs), even in the presence of higher ASA scores, more advanced disease, and increased comorbidities compared to younger counterparts. Despite these baseline differences, the overall postoperative outcomes—including complication rates, hospital stay, and 90-day mortality—were comparable between VEPs and NEP after PSM. These findings align with previous research highlighting the benefits of RATS in elderly patients and reinforcing the notion that chronological age alone should not be a contraindication for surgery.

After PSM, univariate analysis identified ASA score, FEV1, and lobectomy as significant predictors of postoperative complications. Patients with ASA score I, patients treated with lobectomy, and patients with higher values of FEV1 had a lower risk of developing complications. Conversely, patients with an ASA score III had an increased risk of developing complications.

In the multivariate model, after adjusting for confounders and after PSM, the only variables that remained significant independent predictors of complications were FEV1, initial pathological stage I, and lobectomy.

Interestingly, age was never found to be a statistically significant predictor of postoperative complications, neither in the univariate nor in the multivariate analysis, both before and after propensity score matching. This is a particularly encouraging finding, as it supports the concept that chronological age alone should not be considered a contraindication for major pulmonary resection in very elderly patients. The lack of association between age and complications likely reflects the careful preoperative selection of candidates, emphasizing functional status and cardiopulmonary reserve over age per se. It is plausible that age-related frailty and comorbidities, rather than age itself, are the true contributors to postoperative risk. In this regard, in advanced NSCLC it has been reported that the aging of the immune system, defined as immune-senescence does not match with chronological age and may influence the response and survival outcomes upon immunotherapy [22,23]. The effect of the immunological age as a biomarker for complications after surgery remains unknown. Another relevant finding of our study is the independent association between pathological stage I and a significantly lower risk of postoperative complications. This observation underscores the clinical importance of diagnosing lung cancer at an early stage, when the tumor burden is limited and patients are generally in better functional condition. In this context, the implementation and expansion of lung cancer screening programs—particularly low-dose computed tomography (LDCT) in high-risk populations—becomes crucial.

The application of PSM strengthens the reliability of our conclusions, as it minimizes selection bias and allows for a more accurate comparison of surgical outcomes between very elderly and younger patients. Our findings agree with recent studies which also support the use of RATS in elderly patients but lacked a propensity-matched analysis [19]. Our study adds value to the current literature by demonstrating that, even when adjusting for comorbidities and disease severity, RATS remains a safe and effective option in well-selected VEPs.

The results of this study challenge the traditional perception that advanced age should be a primary contraindication for major lung resection. Instead, surgical candidacy should be determined based on functional status, pulmonary function, and systemic health rather than age alone. The following clinical considerations emerge from our findings: RATS is a safe and effective surgical option in elderly patients, with no significant differences in complication rates, hospital stay, or 90-day mortality after PSM.

Our study provides further support for a shift away from age-based surgical exclusion and towards a function-based, individualized approach in elderly patients with lung cancer. By incorporating frailty assessment tools, pulmonary rehabilitation programs, and smoking cessation strategies, we can further refine patient selection and improve surgical outcomes.

The limitations of this study are related to the retrospective nature of the analysis. Another limitation is the difference in the sample size between the two groups, although Propensity Score Matching (PSM) helps to mitigate this issue. The strengths of the paper are the presence of a control group of patients enrolled in the same institution operated by the same surgical teams with the same technique, the use of a modern and validated classification of complications (Clavien–Dindo) and the use of institutional data instead of data from large surgical database.

## 5. Conclusions

This study provides strong evidence supporting the safety and feasibility of RATS in patients aged 80 years and older, demonstrating comparable postoperative outcomes to younger patients after propensity score matching. FEV1, smoking history, and ASA score were the most significant predictors of postoperative complications, while age itself was not an independent risk factor.

These findings advocate for a paradigm shift from age-based surgical decision-making towards function-based patient selection, integrating preoperative pulmonary function, comorbidity burden, and systemic health status into risk stratification. The limitation of the study is related to its retrospective observational nature and because it was performed in only two centers. Future prospective multicenter studies are warranted to validate these results and optimize perioperative management strategies in this growing patient population.

## Figures and Tables

**Table 1:** Descriptive analysis of raw and PSM-corrected preoperative and intraoperative characteristics.

		All Patients				Propensity Score–Matched Patients			
Variable	Category	Overall (*n* = 340)	VEP (*n* = 28)	NEP (*n* = 312)	*p*-Value	Overall (*n* = 97)	VEP (*n* = 26)	NEP (*n* = 71)	*p*-Value
Gender					0.011				1.00
	F	137 (40.3%)	5 (17.9%)	132 (42.3%)		12 (12.4%)	3 (11.5%)	9 (12.7%)	
	M	203 (59.7%)	23 (82.1%)	180 (57.7%)		85 (87.6%)	23 (88.5%)	62 (87.3%)	
Age at surgery (median)		69 (63–75)	81 (80–83)	69 (62–74)	<0.0001	74. (67–80)	81 (80–83)	70 (63–74)	<0.001
BMI		25.2 (23.0–28.0	24.7 (23.5–27.0)	25.2 (22.8–28.0)	0.61	26.2 (24.0–29.6)	24.7 (23.5–27.1)	26.7 (24.2–30.4)	0.078
Number of comorbidities		1 (0–2)	1.5 (1–2)	1 (0–2)	0.0070	1 (1–2)	1.5 (1–2)	1 (1–2)	0.43
ASA score					0.025				0.53
	I	174 (56.1%)	7 (30.4%)	167 (58.2%)		36 (37.1%)	7 (26.9%)	29 (40.9%)	
	II	8 (2.6%)	0 (0.0%)	8 (2.8%)		1 (1.03%)	0 (0.0%)	1 (1.41%)	
	III	124 (40%)	16 (69.6%)	108 (37.6%)		49 (50.5%)	15 (57.7%)	34 (47.9%)	
	IV	4 (1.3%)	0 (0.0%)	4 (1.4%)		3 (3.09%)	0 (0.0%)	3 (4.23%)	
	Missing	30	5	25		8	4	4	
Smoking history					0.053				0.11
	Current smoker	114 (34.7%)	5 (18.5%)	109 (36.1%)		29 (29.9%)	5 (19.2%)	24 (33.8%)	
	Never smoker	68 (20.7%)	4 (14.8%)	64 (21.2%)		16 (16.5%)	2 (7.69%)	14 (19.7%)	
	Past smoker	147 (44.7%)	18 (66.7%)	129 (42.7%)		51 (52.6%)	18 (69.2%)	33 (46.5%)	
	Missing	11	0	11		1	1	0	
Preoperative Chemotherapy					0.152				0.320
	no	315 (93.5%)	27 (100%)	288 (92.9%)		92 (94.85%)	26 (100.00%)	66 (92.96%)	
	yes	22 (6.5%)	0 (0%)	22 (7.1%)		5 (5.15%)	0 (0.00%)	5 (7.04%)	
	Missing	3	1	2					
Preoperative Radiotherapy					0.505				1.000
	No	330 (98.5%)	27 (100%)	303 (98.4%)		96 (98.97%)	26 (100.00%)	70 (98.59%)	
	yes	5 (1.5%)	0 (0%)	5 (1.6%)		1 (1.03%)	0 (0.00%)	1 (1.41%)	
	Missing	5	1	4					
Preoperative Immunotherapy					0.602				1.000
	no	337 (99.1%)	28 (100%)	309 (99%)		96 (98.97%)	26 (100.00%)	70 (98.59%)	
	yes	3 (0.9%)	0 (0%)	3 (1%)		1 (1.03%)	0 (0.00%)	1 (1.41%)	
FEV1%		91 (78–105)	90 (71–112)	91 (78–105)	0.79	89 (76–106)	89 (71–111)	87 (76–104)	0.83
DLCO%		79 (66–96)	86 (71–95)	78 (66–96)	0.48	83 (72–96)	85 (71–94)	80 (73–96)	0.85
Tumor size		2.1 (1.50–3.20)	2.95 (1.90–4.50)	2.0 (1.50–3.0)	0.019	2.50 (1.80–4.30)	2.95 (1.90–4.45)	2.50 (1.80–4.25)	0.66
Side					0.25				0.58
	Left	144 (42.5%)	9 (32.1%)	135 (43.4%)		38 (39.2%)	9 (34.6%)	29 (40.9%)	
	Right	195 (57.5%)	19 (67.9%)	176 (56.6%)		59 (60.8%)	17 (65.4%)	42 (59.2%)	
	Missing	10	1	9		2	1	1	
Surgical procedure									
	Segmentectomy	72 (21.3%)	6 (21.43%)	66 (21.15%)	0.97	17 (17.5%)	6 (23.1%)	11 (15.5%)	0.39
	Lobectomy	248 (73.0%)	18 (64.29%)	230 (73.72%)	0.28	74 (76.3%)	16 (61.5%)	58 (81.7%)	0.040
	Pneumonectomy	8 (2.4%)	2 (7.14%)	6 (1.92%)	0.13	3 (3.1%)	2 (7.7%)	1 (1.4%)	0.17
	Other resections	12 (3.5%)	2 (7.14%)	10 (3.21%)	0.26	3 (3.1%)	2 (7.7%)	1 (1.4%)	0.17
Pathology					0.055				0.049
	Adenocarcinoma	210 (62.3%)	14 (51.9%)	196 (63.2%)		60 (61.9%)	13 (50.0%)	47 (66.2%)	
	Squamous	53 (15.7%)	10 (37%)	43 (13.9%)		22 (22.7%)	10 (38.5%)	12 (16.9%)	
	Other	74 (22%)	3 (11.1%)	71 (22.8%)		15 (15.5%)	3 (11.5%)	12 (16.9%)	
	Missing	3	1	2					
cStage					0.022				0.62
	I	219 (70.6%)	9 (45.0%)	210 (72.4%)		44 (45.4%)	9 (34.6%)	35 (49.3%)	
	II	53 (17.1%)	8 (40.0%)	45 (15.5%)		26 (26.8%)	8 (30.8%)	18 (25.4%)	
	III	31 (10.0%)	3 (15.0%)	28 (9.7%)		16 (16.5%)	3 (11.5%)	13 (18.3%)	
	IV	7 (2.3%)	0 (0.0%)	7 (2.4%)		0 (0.0%)	0 (0.0%)	0 (0.0%)	
	Missing	30	8	22		11	6	5	
pStage					0.76				0.59
	I	200 (63.1%)	16 (61.5%)	184 (63.2%)		46 (47.4%)	15 (57.7%)	31 (43.7%)	
	II	52 (16.4%)	4 (15.4%)	48 (16.5%)		22 (22.7%)	4 (15.4%)	18 (25.4%)	
	III	57 (18.0%)	6 (23.1%)	51 (17.5%)		22 (22.7%)	6 (23.1%)	16 (22.5%)	
	IV	8 (2.5%)	0 (0.0%)	8 (2.7%)		2 (2.06%)	0 (0.0%)	2 (2.8%)	
	Missing	23	2	21		5	1	4	

BMI: Body mass index; ASA: American Society of Anesthesiologists; FEV1: forced expiratory volume in the 1st second; DLCO: diffusing capacity of the lungs for carbon monoxide.

**Table 2:** (**a**) Complications, complications grade, days of postoperative stay, 30 and 90 days mortality in patients submitted to robotic major lung resections. (**b**) Sensitivity analysis for primary outcomes adjusted for surgical procedure.

**(a)**								
	**All Patients**				**Propensity Score–Matched Patients**			
**Primary Outcome**	**VEP**	**NEP**	**Relative Risk, Absolute Mean Difference (95% CI)**	***p*-Value**	**VEP**	**NEP**	**Relative Risk, Absolute Mean Difference (95% CI)**	***p*-Value**
Complications	12 (42.9%)	93 (29.8%)	1.43 (0.91 to 2.27)	0.16	12 (46.2%)	23 (32.4%)	1.43 (0.84 to 2.43)	0.21
**Secondary Outcomes**								
Grade 1 complications	1 (3.6%)	8 (2.6%)	1.39 (0.18 to 10.73)	0.75	1 (3.9%)	2 (2.8%)	1.37 (0.13 to 14.43)	0.8
Grade 2 complications	6 (21.4%)	54 (17.3%)	1.24 (0.59 to 2.62)	0.58	6 (23.1%)	11 (15.5%)	1.49 (0.61 to 3.62)	0.39
Grade 3 complications	4 (14.3%)	20 (6.4%)	2.23 (0.82 to 6.07)	0.12	4 (15.4%)	5 (7%)	2.19 (0.64 to 7.52)	0.21
Grade 4 complications	0 (0.0%)	3 (0.9%)	Not estimable	Not estimable	0 (0.0%)	0 (0.0%)	Not estimable	Not estimable
Postoperative stay (days)	4 (3–5)	4 (3–5)	0.001 (−1.11 to 1.12)	0.99	4 (3–5)	4 (3–6)	0.12 (−1.20 to 1.46)	0.85
30 days mortality	0 (0.0%)	0 (0.0%)	Not estimable	Not estimable	0 (0.0%)	0 (0.0%)	Not estimable	Not estimable
90 days mortality	1 (3.6%)	0 (0.0%)	Not estimable	Not estimable	1 (3.9%)	0 (0.0%)	Not estimable	Not estimable
**(b)**								
	**All Patients**				**Propensity Score–Matched Patients**			
**Primary Outcome**	**VEP**	**NEP**	**Primary Outcome**	**VEP**	**NEP**	**Primary Outcome**	**VEP**	**NEP**
Complications	12 (42.9%)	93 (29.8%)	Complications	12 (42.9%)	93 (29.8%)	Complications	12 (42.9%)	93 (29.8%)

**Table 3:** Univariable analysis of prognostic factors for complications onset.

	All Patients		Propensity Score-Matched Patients	
Variable	OR (CI)	*p*-Value	OR (CI)	*p*-Value
Group (VEP vs. NEP)	1.76 (0.80–3.86)	0.16	1.79 (0.72–4.48)	0.21
Gender M/F	1.87 (1.14–3.08)	**0.013**	1.81 (0.46–7.18)	0.40
BMI	1.02 (0.99–1.05)	0.31	1.02 (0.93–1.12)	0.64
ASA I (ref = 0)	0.65 (0.41–1.03)	0.06	0.36 (0.14–0.92)	**0.032**
ASA II (ref = 0)	1.35 (0.32–5.74)	0.69	0.56 (0.01–54.7)	0.80
ASA III (ref = 0)	1.69 (1.05–2.70)	0.03	2.65 (1.12–6.28)	**0.026**
ASA IV (ref = 0)	2.25 (0.31–16.21)	0.42	0.88 (0.08–10.1)	0.92
Preop Chemotherapy (ref = no)	0.68 (0.24–1.9)	0.4565	0.43 (0.05–3.97)	0.45
Preop Immunotherapy (ref = no)	1.17 (0.11–13.05)	0.8965	0.56 (0.006–54.7)	0.80
Current smoker (ref = never smoker)	4.06 (1.83–9.01)	**0.0006**	1.38 (0.57–3.38)	0.48
Past smoker (ref = never smoker)	3.34 (1.53–7.3)	**0.003**	1.33 (0.58–3.07)	0.50
Tumor size	1.12 (0.98–1.28)	0.088	1.14 (0.90–1.45)	0.28
FEV1	0.97 (0.96–0.99)	**0.0001**	0.97 (0.95–0.99)	**0.024**
DLCO	0.98 (0.96–0.99)	**0.007**	0.98 (0.95–1.01)	0.24
Surgical procedure				
Segmentectomy	0.75 (0.42–1.35)	0.34	2.34 (0.81–6.75)	0.12
Lobectomy	1.04 (0.62–1.74)	0.90	0.33 (0.12–0.85)	**0.022**
Pneumonectomy	1.35 (0.32–5.74)	0.69	0.88 (0.08–10.09)	0.92
Other resections	2.30 (0.72–7.32)	0.16	NA	NA
Clinical Stage				
I (ref = IV)	0.98 (0.19–5.19)	0.98	0.71 (0.31–1.65)	0.43
II (ref = IV)	1.38 (0.24–7.8)	0.72	1.79 (0.72–4.48)	0.21
III (ref = IV)	1 (0.16–6.18)	0.99	0.77 (0.25–2.44)	0.66
Comorbidity	1.27 (0.99–1.63)	0.059	1.16 (0.75–1.79)	0.50
Pathological stage				
I (ref = IV)	1.10 (0.22–5.59)	0.91	0.43 (0.18–1.01)	0.054
II (ref = IV)	1.37 (0.25–7.52)	0.72	2.13 (0.81–5.57)	0.13
III (ref = IV)	1.79 (0.33–9.68)		2.13 (0.81–5.57)	0.13

BMI: body mass index; ASA: American Society of Anesthesiologists; FEV1: forced expiratory volume in the 1st second; DLCO: diffusing capacity of the lungs for carbon monoxide; NA: Not Available.

**Table 4:** Multivariable analysis of prognostic factors for complications onset.

Variable	OR—(Confidence Interval)	Adjusted *p*-Value
Group (VEP vs. NEP)	2.21 (0.93–5.23)	0.073
Never smoker (ref = 0)	0.36 (0.17–0.78)	**0.01**
FEV1	0.98 (0.97–0.99)	**0.002**

VEP: very elderly patient; NEP: non elderly patient; FEV1: forced expiratory volume in the 1st second.

**Table 5:** Multivariable analysis of prognostic factors for complications onset after PSM.

Variable	OR (95% CI)	*p*-Value
Group (VEP vs. NEP)	2.56 (0.85–7.74)	0.095
FEV1	0.97 (0.95–1.00)	**0.025**
pStage I (1 vs. 0)	0.23 (0.08–0.66)	**0.006**
Lobectomy (1 vs. 0)	0.29 (0.09–0.91)	**0.033**

VEP: very elderly patient; NEP: non elderly patient; FEV1: forced expiratory volume in the 1st second.

## Data Availability

We attach the original datasheet to this publication.
